# Social Anxiety and Subjective Quality of Life Among Chinese Left-Behind Children: The Mediating Role of Social Support

**DOI:** 10.3389/fpsyg.2022.836461

**Published:** 2022-03-11

**Authors:** Ying Yang, Xiaozhou Lu

**Affiliations:** School of Educational Sciences, Anshun University, Anshun, China

**Keywords:** social anxiety, social support, subjective quality of life, left-behind children, mediation analysis

## Abstract

The issue of left-behind children has become a key focus in China. In this study, we investigate the mediating role of social support between social anxiety and the subjective quality of life among left-behind children in China (*N* = 379, *M*_*age*_ = 13.65). A total of 710 junior high school students were recruited using clustering random sampling from five middle schools in China and investigated using the Social Anxiety Scale for Children, Social Support Rating Scale for Adolescents, and Inventory of Subjective Life Quality. The results show that social anxiety is negatively associated with social support and subjective quality of life, and social support is positively correlated with subjective quality of life. In addition, social support partially mediates the relationship between social anxiety and subjective quality of life. In conclusion, these findings provide new insights to improve the subjective quality of life of left-behind children. The focus should be on alleviating social anxiety and increasing social support in order to help left-behind children improve their subjective quality of life.

## Introduction

Left-behind children (LBC) is a term that refers to children whose parents (either one or both) leave their hometown for work for at least 6 months (Zhao et al., 2014; Fellmeth et al., 2018). The latest official definition of LBC in China is children under 16 years old with both of their parents at off-hometown work or with one of their parents at off-hometown work and the other incapable of custody (State Council of the PRC, 2016), which is the definition adopted by the current research. The phenomenon of LBC has become a major social problem in China (Ren and Li, 2020). In 2018, the number of LBC under 16 years of age in mainland China was 6.97 million by the end of August while the population aged below 16 was 248.38 million by the end of the year. The approximate proportion of LBC among children under 16 was 1:35.6 (2.81%), which is a small percentage accompanied by a considerable absolute number.

Compared with their peers, who are referred to as non-left-behind children (NLBC), LBC confronted more psychological adversities (Liang et al., 2017; Fellmeth et al., 2018; Wu et al., 2019; Li et al., 2021), were more exposed to moderate abuse, experienced loneliness, attempted suicide when they experienced high levels of loneliness (Chang et al., 2017), and faced a higher suicide risk (Xiao et al., 2019). A study centered on children aged 12–16 showed that the prevalence of mental health problems was 30.8% in NLBC and 43.4% in LBC, with a higher prevalence of psychological symptoms, such as serious psychological distress, panic attacks, depression, and low self-esteem, found amongst LBC than in NLBC (Tang et al., 2018). A meta-analysis showed that the incidence of serious mental health problems in LBC was nearly 2.7 times higher and that LBC’s mental problems included learning anxiety and social anxiety, and were often accompanied by physical symptoms (Wu et al., 2019). Because they are far away from their parents’ care and protection during childhood, left-behind children are more likely to be sexually abused than children who live with their parents (Zhang et al., 2020). Additionally, longitudinal studies revealed that only-mother-migrating LBC showed higher initial depressive symptoms and delinquency levels than only-father-migrating LBC (Lei et al., 2021) and also childhood left-behind experiences significantly impacted the mental well-being of college students (Wu et al., 2021). *More comprehensively*, due to the three issues of lack of family care, family supervision, and family education, these children are prone to learning, mental, and behavioral problems (Chai et al., 2019; Ma et al., 2019). Therefore, the issue of LBC has received increasing attention from the Chinese government (Lei et al., 2018). In recent years, despite the multifaceted policy measures taken by the national government to address the lives, education, and physical and mental health issues of LBC, promoting better development of LBC remains a significant challenge (Wang et al., 2017).

Previous studies have shown that the quality of life of LBC is lower than that of non-left-behind children (NLBC) in China (Huang et al., 2015; Zhu, 2017). Quality of life refers to an individual’s subjective assessment of important needs, aspirations, and goals and the extent to which these needs, aspirations, and goals are subjectively achieved (Frisch, 2000). It is a comprehensive reflection of an individual’s physical and mental state (Hörnquist, 1990). Children’s quality of life affects not only their healthy physical and mental development but also, to a certain extent, the prosperity and wealth of the country, because children represent the future thereof (Wang, 2014; Lu et al., 2016). Therefore, in order to enhance the quality of life of LBC, it is necessary to explore both the potential predictors of quality of life and the mechanisms of action among LBC—especially those in junior high school who are in the critical period of growth about 12 to 15 years old, which covers the first half of stage 5 in Erikson’s theory of psychosocial development (Erikson, 1968; Malone et al., 2016).

Social anxiety (SA) is one of the most prevalent yet neglected psychological problems faced by children (Kashdan and Herbert, 2001; Li et al., 2019). The prevalence rate of SA amongst Chinese rural LBC is 36.1%, which is notably greater than the rate observed amongst their NLBC peers (20.2%) (Li et al., 2019). Social anxiety is defined as a persistent and significant experience of uncomfortable emotions, such as tension and fear, when individuals interact with others in real or imagined social situations (Schlenker and Leary, 1982; Xin et al., 2004). SA not only has a negative impact on the physical and mental health of children (Ren et al., 2017; Peng et al., 2019; Jystad et al., 2021) but may also increase the risk of suicidal ideation in children (Gallagher et al., 2014; Buckner et al., 2017; Lu et al., 2018). Children in junior high school is often a critical period for the development of SA (Gullone et al., 2001), and LBC are more likely to have SA problems due to an unhealthy family structure (Chang and Lu, 2018). A meta-analysis showed that the prevalence among social anxiety of LBC in China (36.1%) was higher than that of their NLBC peers (20.2%) (Li et al., 2019). Some studies have also found that the higher the level of SA in children, the lower their subjective quality of life level (SQOL) (Wang et al., 2010; Xu, 2012). However, to our knowledge, no studies have been conducted in China to specifically explore the relationship between SA and quality of life for this group of LBC.

Furthermore, according to the cognitive-behavioral model of SA (Rapee and Heimberg, 1997; Heimberg et al., 2014), individuals’ negative evaluations of social interaction situations or perceptions of whether these situations are controllable are the main source of SA in individuals. Generally, children with SA will be less active or proactive in interpersonal interactions due to the fear of negative evaluations, neglect, or rejection from others. They will avoid interactions with others to the greatest extent possible, and even try not to ask for help when they have difficulties, which may lead to a lack of social support (SS) in their real lives. Some studies (Wonderlich-Tierney and Vander Wal, 2010; Moghtader and Shamloo, 2019; Barnett et al., 2021) showed that the higher the level of SA of college students, the lower their perceived SS. SS, as an important resource of social characteristics, is an important factor in improving individuals’ perceptions of the controllability of events and enhancing their internal psychological resources (Hill and Hamm, 2019). It also plays an important role in maintaining and promoting individuals’ quality of life (Panayiotou and Karekla, 2013; Wedgeworth et al., 2017; Zhu, 2017). From this, we can infer that SS may play a mediating role between SA and SQOL. However, to our knowledge, no one has yet explored the relationships among SA, SS, and SQOL simultaneously in the same study, especially the mediating role of SS.

In conclusion, LBC represent a social issue of increasing concern in China. This study investigates the relationships among SA and SQOL of LBC in China and the mediating role of SS between SA and SQOL to provide a theoretical basis for improving the SQOL of LBC in China.

## Materials and Methods

### Participants

We used the G*Power software ver. 3.1.9.7 (Faul et al., 2009) to estimate the sample size and found that a minimum of 319 participants would be needed to provide 95% power for a small to moderate correlation (*r* = 0.20, two-tailed test α = 0.05, 1–β = 0.95. A total of 710 students from five junior high schools in Guizhou Province, China, were selected for the survey using the whole-group random sampling method. A total of 710 questionnaires were distributed, and 695 valid questionnaires were returned, with a valid return rate of 97.9%. A total of 379 LBC were screened according to the criteria of “junior high school students under 16 years of age whose parents are both away from home to work, or one parent is away from home to work and the other parent is incapable of custody” as the study subjects. Among them, 178 (46.9%) were single-parent left-behind children and 201 (53.1%) were two-parent left-behind children; 201 (53.0%) were boys and 178 (47.0%) were girls; and 137 (36.1%) were in the seventh grade, 150 (39.6%) were in the eighth grade, and 92 (24.3%) were in the ninth grade. The age of the LBC ranged from 11 to 16 (M_age_ = 13.65, SD_age_ = 0.99). The study was approved by the Ethics Committee of Anshun University, Guizhou, China (approval No. ASU-JYXY-202003). All participants and their legal guardians or next of kin gave written informed consent prior to the survey.

### Measures

#### Social Anxiety Scale for Children

The Social Anxiety Scale for Children was developed by La Greca et al. (1988) and revised by Xin et al. (2004) to assess the level of SA in LBC. The scale includes two dimensions—fear of negative evaluation, and social distress and avoidance —with 10 items (e.g., “I feel shy when I am surrounded by people I don’t know”). The scale is rated using three levels, from 0 (never) to 2 (always). Higher scale scores indicate higher levels of SA. We found the revised Chinese version of the scale to have good reliability and validity, with a Cronbach’s alpha coefficient of 0.82 for the full scale and 0.79 and 0.62 for the two subscales, respectively. Previous studies have also provided evidence of good reliability and validity for this scale (Shi et al., 2019; Ren and Li, 2020). The internal consistency coefficients of the total scale and the two subdimensions in this study were 0.76, 0.72, and 0.60, respectively.

#### Social Support Rating Scale for Adolescents

The Social Support Rating Scale for Adolescents was developed by Ye et al. (2008) with reference to foreign SS scales (Henderson et al., 1980; Sarason et al., 1983). The scale has 17 entries and consists of three dimensions: objective support, subjective support, and support utilization. The scale items are worded as follows: “When in trouble, I usually take the initiative to seek help from others” and “When I have troubles, I will take the initiative to confide in my classmates and friends.” This is a 5-point scale ranging from 5 (agree) to 1 (disagree). The total score of the scale is the sum of the scores of each dimension, and a higher score indicates a higher level of SS. Currently, the scale is widely used in SS studies of adolescents in China and has good reliability and validity (Dai et al., 2010). Previous studies have also provided evidence for the reliability of the scale (Wu et al., 2016). The internal consistency coefficients of the total scale and each subscale in this study were 0.88, 0.76, 0.75, and 0.79, respectively.

#### Inventory of Subjective Life Quality

The Inventory of Subjective Life Quality scale was used to measure the SQOL of LBC (Cheng et al., 1998). The scale includes eight dimensions: peer life, family life, life environment, school life, self-perception, anxiety experience, depressive experience, and somatic emotion. The scale has 52 items and is rated on a 4-point scale, from 1 (none) to 4 (always). The scale items are worded as follows: “Enjoys life at school” and “Enjoys spending time with parents.” Higher scale scores indicate a higher level of SQOL. This scale is standardized, with good reliability and validity for assessing children’s SQOL in China (Dai et al., 2010). Previous studies have provided evidence for the reliability and validity of the scale (Zhu, 2017). The internal consistency coefficient of the full scale was 0.88 (Cheng et al., 1998). The internal consistency coefficient of the scale used in this study was 0.87.

#### Procedures

We recruited participants from five junior high schools in Guizhou Province, China. With the assistance of principals and classroom teachers in several junior high schools, one class from each grade (*7th-9th grades*) in each school was randomly selected, and the questionnaires were distributed and collected on site. The research team administered the test to all participants in a classroom setting using a standardized guide. Prior to administration, participants were informed of the purpose of the study and their right to respond anonymously and to withdraw from the test at any time. Participants were also told to complete the questionnaire independently. Before data collection, all the participants and their legal guardians or next of kin gave written informed consent. The study was approved by the Ethics Committee of Anshun University, Guizhou, China (approval No. ASU-JYXY-202003).

#### Data Analysis

The data were statistically analyzed using SPSS software (Version 25.0). First, a Pearson correlation analysis was used to test the relationship between the SA, SS, and SQOL of LBC. Second, the SPSS macro PROCESS program was used to test the significance of the mediating effects of SS. Third, AMOS 25.0 was used to construct measurement model and structural equation models (SEM) of the variables and analyze their mediating mechanisms. Finally, the “Model Indirect” command in AMOS was used to calculate the standardized indirect effect parameters using 5,000 replicates of bootstrap analysis to test the significance of indirect effects.

In addition, to improve the rigor of the study, we tested for possible common method bias (Podsakoff et al., 2003) prior to conducting statistical analysis. We used Harman’s one-factor test to perform an exploratory factor analysis for all items in the scales prior to rotation. The results showed that a total of 21 factors had eigenvalues greater than 1 and that the variance explained by the first factor was only 13.19%, which is much lower than the critical value criterion of 40% (Podsakoff et al., 2003). Therefore, we can infer that there was no serious common method bias in this study.

## Results

### Descriptive and Correlational Analyses

The correlations and mean total scores of the three scales are shown in Table 1. The results show that SS and SQOL are respectively negatively associated with SA (*r* = −0.17, *p* = 0.001; *r* = −0.27, *p* < 0.001), indicating that LBC reported low levels of SS and SQOL if they had high levels of SA. In addition, the SS positively correlates with SQOL (*r* = 0.47, *p* < 0.001). This meant that the higher the levels of SS in LBC, the higher the levels of SQOL.

**TABLE 1:** Descriptive statistics and correlation analysis among the observed variables.

	1	2	3	4	5	6	7	8
(1) fear of negative evaluation	1							
(2) social avoidance and distress	0.47***	1						
(3) social anxiety	0.90***	0.81***	1					
(4) objective support	−0.06	−0.15**	−0.12*	1				
(5) subjective support	−0.12*	−0.14**	−0.15**	0.57***	1			
(6) support utilization	−0.10	−0.18***	−0.16**	0.59***	0.56***	1		
(7) social support	−0.11*	−0.19***	−0.17**	0.86***	0.81***	0.86***	1	
(8) subjective quality of life	−0.18***	−0.30***	−0.27***	0.41***	0.48***	0.33***	0.47***	1
M	4.68	2.64	7.32	21.91	17.43	20.64	59.98	142.53
SD	2.38	1.79	3.58	4.89	4.06	5.23	12.01	17.11

*P < 0.05, **P < 0.01, ***P < 0.001.

### The Mediating Role Analysis of Social Support With Regression Model

We used Model 4 of SPSS macro PROCESS program for a mediation analysis with SA as the independent variable, SQOL as the dependent variable, SS as the mediators, and used the bias-corrected percentile Bootstrap method for testing the confidence interval (CI) estimates with a repeated sampling of 5,000 to calculate the 95% confidence interval. The results are shown in Table 2. The results show that: (1) SA has a significant negative predictive effect on SQOL and SS (β = −0.27, *t* = −5.48, *p* < 0.001; β = −0.17, *t* = 3.26, *p* < 0.001), respectively; (2) both SA (β = −0.20, *t* = −4.43, *p* < 0.001) and SS (β = 0.44, *t* = 9.72, *p* < 0.00) have significant predictive effects on SQOL. In addition, the Bootstrap method test shows that the total effect size is −0.272 with 95% CI = [−1.776, −0.834]. The indirect effect is −0.075 with a 95%CI [−0.121, −0.026], while the direct effect is −0.20 with 95% CI [−0.1766, −0.834]. In conclusion, SS plays a significant mediating role between SA and SQOL. The mediating effect accounts for 27.574% of the total effect.

**TABLE 2:** Test of the mediating effect of SS in LBC.

Regression equation		Fitting index		Coefficient significance		
Result variable	Predictive variable	*R*	*R* ^2^	*F* value	β value	*t* value
SQOL	SA	0.27	0.07	30.07***	−0.27	−5.48***
SS	SA	0.17	0.03	10.64***	−0.17	−3.26***
SQOL	SA	0.51	0.26	65.99***	−0.20	−4.43***
	SS				0.44	9.72***

SQOL: subjective quality of life;SA: social anxiety; SS: social support ***: P < 0.001.

### Measurement Model

#### The Mediating Role Analysis of Social Support With Structural Equation Models Modeling

Before the mediating role analysis, we firstly used the measurement model to further explore the correlation of variables. An initial test of the measurement model revealed a very satisfactory fit to the data: χ^2^ (4, *N* = 379) = 16.41, *p* < 0.01; RMSEA = 0.071; SRMR = 0.03; GFI = 0.98; AGFI = 0.94; NFI = 0.97; IFI = 0.96; TFI = 0.94 and CFI = 0.98. In addition, the correlation between variables is very significant (*P* < 0.001). The measurement model results show that the data in this study are suitable for SEM modeling.

**FIGURE 1:**
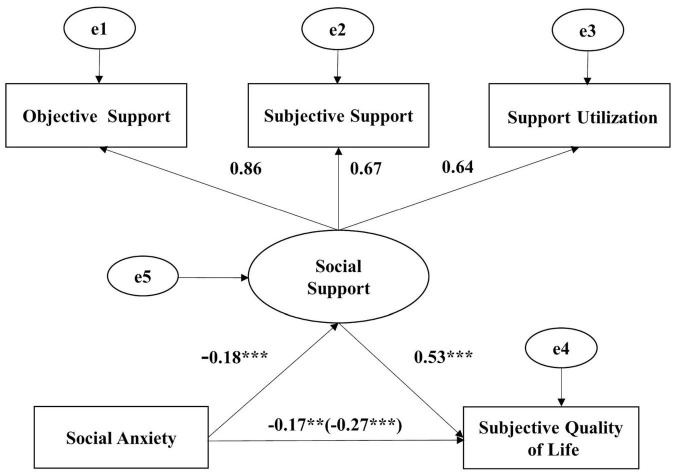
Mediating role of SS between SA and SQOL of LBC. The value in parentheses is the path coefficient of the no direct effect model **p<0.01, ***p<0.001.

Then, we used SEM to construct a mediating effect model, and the results are shown in Figure 1. The mediating effect of SS in Figure 1 indicates that the SA of LBC has a significant negative correlation with SQOL (β = −0.17, *p* = 0.001). SA is significantly negatively associated with SS (β = −0.18, *p* < 0.001) and SS is significantly positively associated with SQOL (β = 0.53, *p* < 0.001).

The fit indices of Model I are χ^2^/df = 1.675, RMSEA = 0.042, AGFI = 0.974, GFI = 0.995, CFI = 0.996, and NFI = 0.990, indicating a high degree of fit of the entire model. Meanwhile, a bootstrap method was used to draw a sample of 5,000 times in AMOS to calculate the potential variable-mediating effects of SS between SA and SQOL. The results show that the total effect size is −0.266 with 95% CI = [−0.369, −0.174], indicating a significant overall effect. The indirect effect is −0.095 with a 95% confidence interval = [−0.124, −0.027], while the direct effect is −0.17 with 95% CI = [−0.288, −0.111]. Thus, the results of the bootstrap analysis of the moment structural equation model also indicate that the SS of LBC mediates the significant association between SA and SQOL. The mediating effect accounts for 35.714% of the total effect.

In conclusion, we used regression model and structural equation model to test the mediating effect of SS. The results of two different methods both show that SS plays a significant intermediary role between SA and SQOL.

## Discussion

This study is the first to examine the relationship between SA and SQOL in Chinese LBC and the mediating role of SS. First, the findings show that SA is negatively associated with SQOL in junior high school students. Specifically, the higher the level of SA in LBC, the lower its SQOL level. This is consistent with the results of previous studies (Wang et al., 2010; Xu, 2012). Compared to NLBC, LBC receive less care and instruction from their parents in life, learning, and interpersonal skills (Ren et al., 2017), because they are separated from them so much. Zou et al. (2021) pointed out that adolescents with good family support, parent-child communication, family cohesion, and parental supervision had lower levels of social anxiety; however, for left-behind middle school students, the family functions mentioned above were missing to some extent. According to Rogers, individuals who do not receive unconditional, positive, or conditional attention and guidance from their parents during their growth will gradually develop self-incongruence (Rogers, 1957; Wouters et al., 2018)—that is, doubt themselves, evaluate themselves based on other people’s values, and develop feelings of inferiority, as well as becoming timid and anxious (Yang, 2001). Moreover, it was revealed in longitudinal research that left-behind experiences in childhood had a negative effect on mental well-being in college students (Wu et al., 2021). In addition, SA self-presentation theory (Leary and Jongman-Sereno, 2014) suggests that SA stems from concerns about negative evaluations by others, especially peers; this is a particular concern for adolescents (Barzeva et al., 2020; Chiu et al., 2021). More importantly, the social anxiety of retained junior high school students may be related to Chinese culture. Chinese culture emphasizes collectivism, etiquette, shame, and face and promotes the social interaction norms of modesty, introversion, and unobtrusiveness (Fang, 2012), which may be linked to the fact that retained junior high school students are especially concerned about their performance in interpersonal interactions, desire recognition from others, fear negative evaluations in social interactions, and are prone to feel shame at the slightest indiscretion and thus avoid interpersonal interactions. A study by Fang (2013) found that the shame of LBC significantly positively predicted their SA. In general, adolescents with SA will be less active or proactive in interpersonal interactions, will minimize their willingness to communicate and cooperate with others, will be reluctant to ask for help when they have problems, and will experience more loneliness and depression (Hamilton et al., 2016; Li et al., 2020; Wang and Yao, 2020; Chiu et al., 2021). This directly affects their subjective perception of their quality of life. The findings suggest that alleviating SA in LBC may be an important way to improve their quality of life.

Second, the findings of this study suggest a significant positive correlation between SS and SQOL. For LBC, the higher the levels of SS, the higher the levels of SQOL. This finding is similar to those of previous studies (Panayiotou and Karekla, 2013; Wedgeworth et al., 2017; Zhu, 2017). Furthermore, the result is consistent with the causal model of SS (Ren and Li, 2020), which emphasizes that increasing SS improves the SQOL of LBC. Theoretically, the presence of SS may help to enhance individuals’ psychological well-being and subjective happiness (Kondrat et al., 2018). According to SS theory, the stronger the SS network an individual has, the better they can cope with various challenges from the environment and the easier it is to adapt to different environments (Fu et al., 2012) as they experience more positive emotions (Zhou et al., 2013).

In recent years, the issue of LBC in China has received widespread attention from all sectors of society and they can receive material or psychological and emotional support from their families, communities, schools, or other sources. Therefore, LBC can use their acquired SS as a positive coping strategy to adapt to their environment and combat conflicting life events (Davis and Brekke, 2014), ultimately enhancing their satisfaction with their living environment, and their physical and mental health. The findings of this study reveal that the SS system of LBC is an important condition that affects their SQOL, and improving this system can help to enhance their SQOL.

Third, and most importantly, the findings of this study demonstrate that SS mediates the relationship between SA and SQOL. LBC with high levels of SA try to avoid communicating and interacting with classmates, teachers, and parents due to the fear of negative evaluations from others in interpersonal interactions. They are reluctant to ask for help even when they encounter difficulties, which to a certain extent reduces the acquisition, perception, and utilization of SS and, at the same time, reduces the internal psychological resources that can alleviate SA from the perspective of SS (Wang et al., 2014). Thus, compared to LBC with low SA levels and high SS levels, LBC with high levels of SA and low levels of SS may experience more anxiety and depression, which reduces their perceived satisfaction with quality of life. Conversely, LBC with low levels of SA have more internal psychological resources to reduce the negative effects of SA through more interpersonal interactions with others. This can lead to more SS, which can help to change negative evaluations of social situations or increase perceptions of the controllability of the interpersonal environment, thereby increasing self-esteem and promoting more positive emotional experiences (Wedgeworth et al., 2017), which in turn increases perceptions of quality of life satisfaction. In summary, SA in LBC can have an indirect effect on SQOL through SS, which further suggests the importance of increasing SS levels in LBC to alleviate their SA and enhance their SQOL.

Finally, due to the limitations of the study, the results of this study should be interpreted with caution. First, all data in this study were derived from LBC self-reports of SA, SS, and SQOL, and qualitative indicators of these variables obtained through methods such as interviews were lacking. Second, this study utilized a cross-sectional design for LBC, and although it provides proof of the relationship between SA, SS, and SQOL, it remains unclear exactly how these relationships change dynamically. Third, there may be other mediating variables, such as self-esteem, coping style, and personality, in addition to SS, between SA and SQOL in LBC. Future studies could further explore these issues. Finally, further studies should test whether there are different mechanisms underlying the association between SA and SQOL in both LBC and Non-LBC.

## Conclusion

To our knowledge, so far, no study has examined the relationship between SA, SS, and SQOL in Chinese LBC. The results of the study support the relationship between SA and SQOL. Furthermore, SS is found to play a mediating role in this relationship. The findings of this study may provide new perspectives on how to enhance the SQOL of LBC in China. Given the mediating role of SS, the level and intensity of SS for LBC should continue to be increased to improve the SQOL of LBC reporting high levels of SA. In addition, given the direct impact of SA on SQOL, interventions aimed at mitigating SA (e.g., social interaction skills training and changing negative evaluations of social interaction situations) should be implemented to improve the SQOL of LBC.

## Data Availability Statement

The original contributions presented in the study are included in the article/supplementary material, further inquiries can be directed to the corresponding author/s.

## Ethics Statement

The studies involving human participants were reviewed and approved by School of Educational Sciences, Anshun University. Written informed consent to participate in this study was provided by the participants’ legal guardian/next of kin.

## Author Contributions

YY proposed the research idea and design, collected and analyzed the data, wrote and reviewed the draft of the manuscript, and approved the final draft. XL justified the research idea and design, collected data, reviewed the draft manuscript, and approved the final draft. Both authors contributed to the article and approved the submitted version.

## Conflict of Interest

The authors declare that the research was conducted in the absence of any commercial or financial relationships that could be construed as a potential conflict of interest.

## Publisher’s Note

All claims expressed in this article are solely those of the authors and do not necessarily represent those of their affiliated organizations, or those of the publisher, the editors and the reviewers. Any product that may be evaluated in this article, or claim that may be made by its manufacturer, is not guaranteed or endorsed by the publisher.
